# The Effects of Milking and Cleaning Procedures on the Quality and Microbiome of Raw Goat Milk

**DOI:** 10.3390/foods14203563

**Published:** 2025-10-20

**Authors:** Alyssa Thibodeau, Eiseul Kim, Seung-Min Yang, Lisbeth Goddik, Hae-Yeong Kim, Si Hong Park

**Affiliations:** 1 Department of Food Science and Technology, Oregon State University, Corvallis, OR 97331, USA; alyssa.thibodeau@oregonstate.edu (A.T.); eskim89@khu.ac.kr (E.K.); ysm9284@gmail.com (S.-M.Y.); lisbeth.goddik@oregonstate.edu (L.G.); 2 Department of Food Science and Biotechnology, Kyung Hee University, Yongin 17104, Republic of Korea; hykim@khu.ac.kr

**Keywords:** raw goat milk, quality, microbiome, food safety

## Abstract

The rising popularity of raw goat milk has heightened concerns about its safety. This study examined how differences in milking and cleaning practices influence the quality and microbiota of goat milk from small-scale Oregon farms during July and August. Milk quality was assessed through somatic cell counts (SCCs) and components, while microbiota was evaluated using viable counts and 16S rRNA sequencing. Sequencing revealed a diverse microbial community, dominated by genera such as *Staphylococcus*, *Escherichia-Shigella*, and *Pseudomonas*, with pathogenic taxa like *Salmonella* and *Campylobacter* largely absent or detected at negligible levels. Alpha diversity varied significantly among sample types but not across farms, and beta diversity indicated considerable dissimilarity in microbial composition. Importantly, regression models identified significant associations between hygiene practices and bacterial abundance: the absence of glove use and hand sanitation was linked to increased levels of *Escherichia-Shigella*, *Kocuria*, *Enterococcus*, and *Corynebacterium*, while the use of bleach-chlorhexidine sanitizer was associated with higher *Deinococcus*. These findings highlight the role of rigorous hygiene protocols in shaping the microbiota of raw goat milk and emphasize the need for targeted practices to minimize contamination risks.

## 1. Introduction

Milk provides an ideal environment for microbial growth due to its nearly neutral pH, high water activity, and rich nutrient profile [1]. Although milk within the udder of a lactating animal is considered sterile, it becomes exposed to contaminants from the air, milking equipment, or residual microbes on the teat surface during milking [2,3]. This exposure, combined with milk’s nutrient-rich nature, facilitates rapid microbial growth and potential degradation, influenced by factors such as feeding practices and animal handling during milking [4]. Effective pre- and post-milking procedures are essential for maintaining the quality and microbiological safety of raw milk.

The demand for goat dairy products has been rising globally, with goat milk production projected to increase by more than 50% by 2030 [5,6]. In the U.S., over 35,000 farms were raising dairy goats in 2017, and the vast majority were small-scale operations, with nearly all having fewer than 500 goats and more than half maintaining fewer than 10 [7]. As consumer interest in goat milk and derived products grows, often driven by perceptions of health benefits, ensuring product safety and consistency has become increasingly important [8].

Raw goat milk harbors a diverse microbiota, including lactic acid bacteria such as *Lactobacillus* and *Lactococcus*, along with genera like *Pseudomonas* and *Streptococcus* [9,10]. The safety and quality of goat milk are shaped by animal health, hygiene during milking, and equipment sanitation [3,11]. Previous studies have shown that poor milking hygiene and inadequate cleaning of equipment can elevate bacterial contamination, reducing both shelf life and safety [12,13]. Goat milk quality is commonly assessed by parameters such as chemical composition, somatic cell count (SCC), microbial load, and sensory properties [14,15]. These attributes vary according to breed, lactation stage, and milking time, underscoring the complexity of maintaining consistent quality [16,17,18,19].

Recent research has highlighted the nutritional and functional attributes of goat milk, including its digestibility, lower allergenicity, and probiotic potential [20,21]. Advances in microbial profiling tools such as 16S rRNA sequencing and metagenomics have revealed the complexity of the goat milk microbiome and its implications for food safety and functionality [22,23]. Studies have also investigated pathogen prevalence and antimicrobial resistance in raw goat milk, emphasizing the need for rigorous hygiene and monitoring [22]. In practice, small-scale producers typically adopt basic but critical measures such as pre- and post-milking teat sanitation, cleaning equipment with hot water and sanitizers, and rapid cooling of milk [24]. However, these approaches are largely adapted from bovine systems and may not fully suit goats, highlighting the need for species-specific best practices.

Despite the perception of raw milk as “risky,” consumer interest continues to grow, particularly among small-scale farm markets. In the U.S., the Food and Drug Administration (FDA) prohibits interstate raw milk sales, but individual states establish their own rules [25]. In Oregon, for instance, farms with fewer than nine goats may sell raw milk directly on-site under defined conditions (O.R.S. § 621.012 and O.R.S. § 621.117) [26]. Yet, small producers often lack the resources to systematically validate their practices, and the absence of goat-specific standards creates inconsistencies. This study therefore aims to evaluate how variations in milking and cleaning practices influence the microbiome and overall quality of raw goat milk, with the goal of identifying best practices that minimize contamination while ensuring safe, high-quality production.

## 2. Materials and Methods

### 2.1. Study Design

To participate in this study, farms had to meet two criteria: (1) milking fewer than 9 goats and (2) producing raw milk for direct human consumption. Small-scale goat farmers and homesteaders within a 60-mile radius of Corvallis, Oregon, who met these criteria were invited to join. Four farms (A, B, C, and D) were recruited, and samples were collected twice a week during the 2nd, 3rd, and 4th weeks of July and August 2021 (Table 1). This involved a total of six visits to each farm, resulting in 24 visits overall. During the initial visit to each farm, a comprehensive assessment was conducted to document operating practices and logistics. Information gathered included milking and cleaning procedures, frequency and types of cleaning agents, milking frequency (once or twice daily), maintenance routines for the milking parlor and barn, and details about milking equipment replacement. Additionally, farm-specific details such as goat breed(s), stage of lactation, parity, and age were recorded. To qualitatively assess hygiene conditions in a consistent manner, a structured visual assessment was performed by the same trained researcher during farm visits. A checklist was used to evaluate barn/parlor layout, barn/parlor cleaning routine, glove or hand sanitizer use, udder preparation steps, and equipment cleaning practices. Each criterion was coded on a 0–2 scale as described in Table 2.

### 2.2. Sample Collection

During each visit, raw milk and environmental samples were collected. Milk samples were taken in quadruplet, using 50 mL conical tubes, while environmental samples were collected in duplicate using PUR-Blue Swabs (World Bioproducts LLC, Woodinville, WA, USA). Environmental samples included swabs from the bulk tank interior surface, teat cup liners, and the milking stanchion. Each area (approx. 10 × 10 cm) was swabbed for ~10 s using sterile cotton swabs moistened with buffered peptone water. Sampling was performed immediately before milking under ambient barn conditions, and swabs were placed into sterile tubes and transported on ice to the laboratory for microbiological analysis. All collected samples were immediately stored in an ice-filled cooler and transported to the lab. Samples were either processed the same day or stored in a −20 °C freezer for further analysis.

### 2.3. Microbial Enumeration

Raw milk samples were collected once a week for three consecutive weeks in both July and August from each farm and processed on the day of collection. Microbial enumeration was performed according to internationally recognized methods [27,28,29,30,31]. Samples were serially diluted in sterile deionized water. For the preparation of the first decimal dilution, 1 mL of raw milk was added to 9 mL of sterile deionized water. For each diluted sample, 1 mL was plated on aerobic plate count (APC, Peel Plate AC, Charm Sciences Inc., Lawrence, MA, USA), coliform count (CC, Peel Plate CC, Charm Sciences Inc.), and yeast and mold count plates (YM, Peel Plate YM, Charm Sciences Inc.). The APC plates were incubated at 35 °C for 24 h, the CC plates at 35 °C for 24 h, and the YM plates at 30 °C for 72 h. Colony-forming units (CFU/mL) were enumerated from triplicate plates for each dilution, averaged to obtain a representative value, and log_10_-transformed prior to statistical analysis.

### 2.4. SCC and Milk Component Analysis

SCC and milk component analysis were conducted on the same raw milk samples that were also used for microbiological counts. To prevent microbial growth and ensure sample integrity, raw milk was immediately treated with a bronopol and natamycin preservative tablet (Broad Spectrum Microtabs II, Norwood, MA, USA) and stored at 4 °C for up to one week before analysis. For somatic cell counts, 50 mL of raw milk was used with the flow cytometry (SomaScope Smart, Delta Instruments BV, Drachten, The Netherlands). Milk components, including true protein, fat, lactose, and blood non-esterified fatty acids (NEFA), were analyzed using 50 mL of raw milk with the fourier-transform infrared spectroscopy (FTIR) milk analyzer (LactoScope, Delta Instruments BV). Each SCC measurement was performed in duplicate, and the two values were averaged to yield a representative value per week. SCC values were log_10_-transformed prior to statistical analysis.

### 2.5. Milk Microbiome Analysis

#### 2.5.1. DNA Extraction

For raw milk samples, 1 mL of milk was homogenized by vortexing and then centrifuged at 13,000× *g* for 15 min at 4 °C. The supernatant was discarded, and the pellet was washed with 500 µL of phosphate-buffered saline (PBS). DNA was then extracted from the pellet using the Qiagen Power Foods DNA extraction kit (Qiagen, Hilden, Germany), following the manufacturer’s instructions. Environmental samples (bulk tank, teat cup liner, and milking stanchion) were collected in 1 mL increments. The samples were then centrifuged at 1000× *g* for 2 min at room temperature. The pellets were processed for DNA extraction using the Qiagen Blood & Tissue Kit (Qiagen) with the protocol for Gram-positive bacteria. DNA concentrations from both raw milk and environmental samples were measured using a Qubit 3.0 fluorometer (Thermo Fisher Scientific, Waltham, MA, USA).

#### 2.5.2. Microbiome Sequencing

Following DNA extraction, an amplicon library of the V4 region of the 16S rRNA gene was created for both milk and environmental samples using the protocol described by previous study [32]. DNA fragments were amplified using AccuPrime™ Pfx SuperMix (Thermo Fisher Scientific), and successful amplification was confirmed via gel electrophoresis on a 1.5% agarose gel. PCR products were normalized with the SequalPrep™ Normalization Plate Kit (Thermo Fisher Scientific), following the manufacturer’s instructions. A 5 µL aliquot of each normalized sample was pooled to create a sequencing library, which was then sequenced at the Oregon State University Center for Quantitative Life Sciences (CQLS) using an Illumina MiSeq Reagent Kit v2 (2 × 250 bp, 500 cycles; Illumina Inc., San Diego, CA, USA).

### 2.6. Statistical Analysis

#### 2.6.1. Statistical Analysis of APC, CC, YM, and SCC Data

Due to the small sample size, normality and homogeneity of variance were not formally tested, and non-parametric statistical methods were applied. Differences among farms were assessed using the Kruskal–Wallis test, followed by Dunn’s post hoc test. Multiple testing correction was applied using both the Bonferroni and Benjamini–Hochberg (FDR) procedures. All analyses were performed in R version 4.3.2 using the Dunn test and FSA packages. Statistical significance was defined as *p* < 0.05.

#### 2.6.2. Metagenomic Analyses

Raw sequencing data were analyzed using QIIME 2 (version 2021.4), the latest stable release at the time of analysis (2021–2022) [33]. Demultiplexed paired-end reads were processed with the DADA2 plugin for quality filtering, denoising, and chimera removal. Taxonomy was assigned using the q2-feature-classifier plugin with a Naive Bayes classifier pre-trained on the SILVA 138 database. Alpha diversity was assessed with the Shannon index, and beta diversity with Bray–Curtis dissimilarity, followed by Analysis of Similarities (ANOSIM) to compare group-level differences. Relative abundances of dominant genera were calculated from the feature table using QIIME 2 and further processed in Microsoft Excel. Differences in genus-level abundance among farms were analyzed using the Kruskal–Wallis test with Dunn’s post hoc test and multiple testing correction (Bonferroni and Benjamini–Hochberg).

To examine associations between milking hygiene practices and bacterial genera, linear regression analyses were conducted in R (stats package). The relative abundance (arcsine square-root transformed) of selected genera was modeled as the outcome variable, with predictor variables including glove use, hand sanitation between animals, and sanitizer type. All categorical predictors were coded as factors, with reference levels set to “Yes” (glove use, hand sanitation) and “Bleach-Iodine” (sanitizer type). Farm identity was treated as a blocking variable and checked in sensitivity analyses. Model assumptions (linearity, normality of residuals, and homoscedasticity) were verified through residual diagnostics. Statistical significance was defined as *p* < 0.05.

## 3. Results

### 3.1. Cleaning Intensity Based on Farm Assessment

Based on the structured visual assessment, hygiene practices were coded for each farm according to barn/parlor layout, barn/parlor cleaning, glove or hand sanitizer use, udder preparation, and equipment cleaning (Table 3). Farm A consistently received the highest codes across categories, with semi-enclosed facilities, regular sweeping and mopping, consistent glove use, multi-step udder preparation using iodine and bleach, and thorough equipment sanitation with hot water and bleach. Farm B scored lowest overall, with a separate enclosed parlor. Although barn/parlor cleaning was performed regularly, this farm showed limited overall hygiene due to the absence of glove or sanitizer use, minimal udder preparation (wipes only), and inconsistent equipment cleaning with lukewarm water and short contact times. Farm C showed mixed practices: although the barn cleaning score was lowest (occasional sweeping only), this farm employed moderate udder preparation with wipes and dip, occasional glove or sanitizer use, and thorough equipment cleaning through a dishwasher cycle with boiling hot water. Farm D achieved high codes for barn/parlor cleaning and regular soap-bleach steps in udder preparation but did not use gloves or hand sanitizers and relied on short contact times for equipment sanitation.

### 3.2. Microbiological Analyses

**Figure 1:**
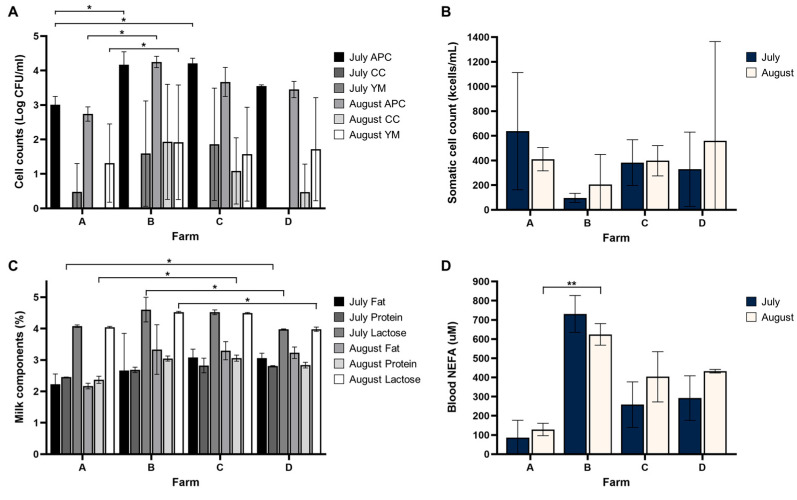
Microbiological and quality assessments of raw milk samples collected from farms A, B, C, and D in July and August. (**A**) Plate counts, including aerobic plate count (APC), coliform count (CC), and yeast and mold (YM), for each farm. (**B**) Somatic cell counts (SCC), showing variations between July and August for each farm. (**C**) Milk components, including true protein, fat, and lactose levels. (**D**) Blood non-esterified fatty acids (NEFA) concentrations in raw milk. Error bars represent the standard deviation of triplicate measurements. Asterisks indicate significant differences between farms based on Kruskal–Wallis tests followed by Dunn’s post hoc test with multiple comparison adjustment. *, *p* < 0.05; **, *p* < 0.01.

Mean and standard deviation values for APC, YM, and CC are illustrated in Figure 1A for both July and August. Nonparametric analysis (Kruskal–Wallis with Dunn’s post hoc test) indicated significant differences in APC between farms in July (*p* = 0.025) and August (*p* = 0.031) (Table 4). In July, farm A had significantly lower APC compared with farms B and C. In August, APC in farm A was again significantly lower than in farm B (*p* = 0.010). No significant differences were observed in YM and CC in July. In August, YM showed a marginal trend (*p* = 0.072), and post hoc comparison revealed a significant difference only between farms A and B (*p* = 0.036). CC values did not differ among farms in August. The relatively large standard deviations observed in some groups likely reflect inherent biological variability among farms and animals, as well as week-to-week variation within each month, since three weekly samples were collected.

### 3.3. Results of SCC and Milk Component Analysis

Mean and standard deviation values for SCC, milk components, and blood NEFA are illustrated in Figure 1 for both July and August. SCC did not differ significantly among farms in July (*p* = 0.161) or August (*p* = 0.578) (Table 4). Pairwise comparisons also confirmed no significant differences. For milk fat, no significant differences were detected in either July (*p* = 0.228) or August (*p* = 0.099). Blood NEFA concentrations did not differ in July (*p* = 0.459) but there were significantly different in August (*p* = 0.024), with farm A showing lower values than farm B (*p* = 0.007). Protein content varied among farms in July (*p* = 0.045), with farm A lower than farm D (*p* = 0.020). In August, protein also differed (*p* = 0.024), with farm A lower than farm C (*p* = 0.020). Lactose content showed significant variation in both July (*p* = 0.024) and August (*p* = 0.030). In July, farms B and D differed marginally (*p* = 0.020), and in August, the difference between farms B and D was significant (*p* = 0.028).

### 3.4. Microbial Diversity

#### 3.4.1. Alpha Diversity

**Figure 2:**
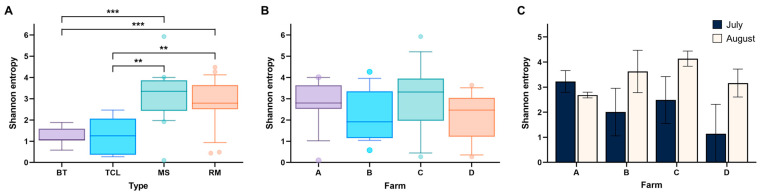
Alpha diversity (Shannon index) of microbial communities across various sample types, locations, and collection periods. (**A**) Alpha diversity across different sample types: bulk tank (BT), teat cup liner (TCL), milking stanchion (MS), and raw milk (RM), highlighting variation in microbial richness and evenness among these categories. (**B**) Alpha diversity comparison between locations, including all samples (raw milk and environmental samples combined). (**C**) Alpha diversity of raw milk samples collected in July and August, illustrating seasonal variations in microbial diversity. Error bars represent standard deviations, indicating variability within each category. Asterisks indicate significant pairwise differences among sample types based on Kruskal–Wallis tests followed by Dunn’s post hoc test with multiple comparison adjustment. **, *p* < 0.01; ***, *p* < 0.001.

Alpha diversity differed significantly among sample types (*p* < 0.0001). Milking stanchion and raw milk samples showed significantly higher diversity than bulk tank samples (*p* < 0.001). Teat cup liner samples did not differ significantly from bulk tank samples but exhibited lower diversity than both milking stanchion and raw milk samples (*p* < 0.01). No significant difference was observed between milking stanchion and raw milk samples (*p* = 0.509) (Figure 2A). In contrast, alpha diversity did not significantly differ among farms (*p* = 0.112) (Figure 2B). Although Farm C tended to show higher diversity and farm D lower, none of the pairwise comparisons reached statistical significance after multiple testing correction (*p* > 0.058). We also compared the alpha diversity of raw milk samples between months. No significant differences were found among farms in either July (*p* = 0.10) or August (*p* = 0.079). In July, farm D tended to show lower diversity than farm A (*p* = 0.052), and in August, farm C tended to be more diverse than farm A (*p* = 0.052), but these differences were not statistically significant after multiple testing correction (Figure 2C).

#### 3.4.2. Beta Diversity

Beta diversity was initially evaluated using pairwise comparisons, revealing statistically significant differences among all samples. However, these differences did not distinctly separate the farms. To further analyze beta diversity, an ANOSIM test was performed to assess the similarity or dissimilarity of the microbiota among samples. The Unweighted UniFrac metric was used, which measures the fraction of unshared microbial groups between samples. R-values closer to 1 indicate greater dissimilarity, while values closer to 0 indicate greater similarity [34]. For both July and August, R-values for the raw milk samples ranged from 0.44 to 0.67, suggesting considerable dissimilarity in microbial communities, though the data were not strong enough to draw definitive conclusions.

### 3.5. Taxonomic Analysis

#### 3.5.1. Farm-Level Microbiota Composition

**Figure 3:**
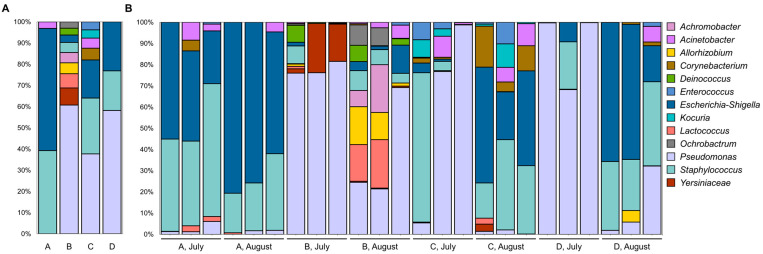
Microbiome composition across different farms (A, B, C, and D) based on relative abundance at the genus level. (**A**) Microbial community structure in combined samples (raw milk and environmental samples) from each farm, illustrating the overall microbial diversity and distribution across different farm types. (**B**) Microbial community structure specifically in raw milk samples from each farm, highlighting the dominant genera present in raw milk alone. Each color represents a different genus, and the varying heights of color blocks indicate the relative abundance of each microbial group within the samples.

A total of 611 taxonomic groups were identified at the genus level across all raw milk and environmental samples. Farm A’s microbiota was dominated by *Staphylococcus* and *Escherichia*, with no presence of *Pseudomonas*. In contrast, farm B had a higher abundance of *Pseudomonas* along with notable amounts of *Yersiniaceae*, *Lactococcus*, and other taxa. Farm C exhibited significant proportions of *Staphylococcus* and *Escherichia*, with smaller amounts of *Acinetobacter* and *Corynebacterium*. Farm D was characterized by a large proportion of *Pseudomonas* and *Staphylococcus*, with fewer additional taxa (Figure 3A).

#### 3.5.2. Raw Milk Samples

When focusing specifically on raw milk samples, the dominant microbial groups were *Staphylococcus*, *Escherichia*-*Shigella*, and *Pseudomonas*, showing considerable variation between samples (Figure 3B). *Pseudomonas* was particularly abundant in certain samples from farms B and D, whereas *Staphylococcus* and *Escherichia*-*Shigella* were prevalent across multiple raw milk samples from all farms. Some taxa, like *Yersiniaceae* and *Lactococcus*, appeared sporadically with low relative abundance. Importantly, the pathogenic genus *Salmonella* was not detected in any of the samples, and *Campylobacter* was found in only one milk sample from farm B in August, with a very low relative abundance of 0.014%. The microbial composition varied distinctly between farms.

#### 3.5.3. Genera of Interest

**Figure 4:**
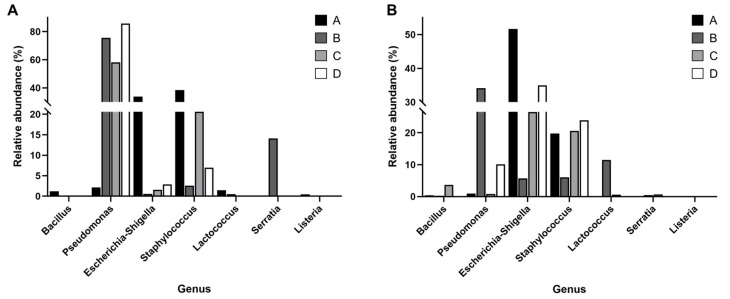
Relative abundance of seven major pathogenic bacteria identified in raw milk samples from different farms during (**A**) July and (**B**) August. Each bar represents the percentage of a specific pathogenic bacterial group within the sample, illustrating seasonal and farm-level variations in microbial composition.

Seven genera of interest for comparison included *Bacillus*, *Pseudomonas*, *Escherichia*-*Shigella*, *Staphylococcus*, *Lactococcus*, *Listeria*, and the family *Yersiniaceae* (Figure 4). Except for *Lactococcus*, all these groups are considered non-beneficial microorganisms. Among the bacterial genera analyzed, *Pseudomonas* and *Escherichia*-*Shigella* exhibited significant differences in relative abundance across farms during August (*p* = 0.048 and 0.038, respectively). In August, *Escherichia*-*Shigella* abundance was significantly higher in farm A than in farm B (*p* = 0.014). While *Pseudomonas* abundance showed a significant overall difference, post hoc comparisons revealed a marginal difference between farms C and B (*p* = 0.052). In July, *Escherichia*-*Shigella* and *Pseudomonas* showed a trend toward significance (*p* = 0.066 and 0.086, respectively), but no pairwise comparisons reached statistical significance after correction. Other genera such as *Bacillus*, *Staphylococcus*, *Lactococcus*, *Yersiniaceae*, and *Listeria* did not show statistically significant differences among farms at either time point (*p* > 0.05). The Kruskal–Wallis test could not be performed for *Listeria* in August due to uniform values across groups.

### 3.6. Association Between Milking Hygiene Practices and Bacterial Abundance

Linear regression models were constructed to assess the association between hygiene-related practices and the relative abundance of dominant bacterial genera. The reference level (intercept) for all categorical variables was set as glove use = “yes”, hand sanitation = “yes”, and sanitizer type = “bleach iodine”. Among the taxa analyzed, *Escherichia*_*Shigella* showed the strongest association (R^2^ = 0.526, *p* = 0.002) (Table 5). Specifically, its intercept was highly significant (*p* = 9.32 × 10^−7^). *Deinococcus* abundance was significantly influenced by both the absence of hand sanitation (*p* = 0.005) and the use of bleach chlorhexidine sanitizer (*p* = 0.005) (R^2^ = 0.428, *p* = 0.010). *Kocuria* (R^2^ = 0.435, *p* = 0.009) and *Enterococcus* (R^2^ = 0.365, *p* = 0.026) were both significantly elevated in conditions where gloves were not used and hand sanitation was not performed. Likewise, *Corynebacterium* (R^2^ = 0.351, *p* = 0.032) showed significant increases under the same conditions (*p* = 0.026 and *p* = 0.010, respectively). *Staphylococcus* exhibited a significant intercept (*p* = 5.59 × 10^−5^). The remaining taxa did not show statistically significant associations (*p* > 0.05).

## 4. Discussion

This study aimed to investigate how variations in milking and cleaning practices influence the quality and microbiome of raw goat milk. The findings demonstrate that farms with more rigorous cleaning protocols generally have lower counts of spoilage-specific microorganisms, which aligns with the research objective of identifying best practices for minimizing contamination.

APC, CC, and YM results showed that farms implementing multiple kill steps and frequent sanitization had fewer microbial contaminants. For example, farm A, which followed the most intensive cleaning procedures, including specified sanitizer concentrations, frequent milk parlor cleaning, and regular sanitation of equipment, consistently had coliform counts below the detection limit (<10 CFU/mL). This supports the established link between thorough hygiene practices and lower microbial contamination, as noted by Pantoja et al. (2011), who emphasized the importance of udder hygiene and bulk tank sanitation [35]. Farm D, despite not using precise sanitizer concentrations or gloves, had comparable microbial counts to farm A, suggesting that frequency and method of cleaning may outweigh specific product usage in some cases. Interestingly, despite overall rigorous cleaning, farm A exhibited significantly higher YM levels than farm B in August (*p* = 0.036), suggesting that fungal contamination may be influenced by environmental or equipment-related factors. As Vacheyrou et al. (2011) noted, fungal species in milk are often linked to the milking environment [36]. Farm A’s less frequent replacement of equipment, such as gaskets and hoses, may have contributed to the persistence of YM, despite thorough cleaning practices. This highlights the importance of regularly replacing equipment, in addition to cleaning. Furthermore, the absence of acid or caustic wash steps at farm A suggests that incorporating these agents could help further reduce yeast and mold levels [37].

Although no statistically significant differences in SCC were observed among farms, considerable week-to-week fluctuations were detected, often coinciding with reported health issues in the herds. This observation is consistent with previous studies in goats, which emphasized that SCC are more strongly influenced by herd health status, stage of lactation, and parity than by milking hygiene alone [38,39]. Similar findings have been reported in dairy cows, where SCC variation largely reflected udder health and physiological factors rather than external management practices [40]. In our study, extreme weekly values likely contributed to the even distribution of mean SCC across farms, underscoring the multifactorial nature of this trait. Thus, while SCC remains an important indicator of udder health, its interpretation requires consideration of multiple interacting factors. In contrast, milk composition parameters such as protein and lactose showed clearer farm-level differences, with farm A consistently exhibiting lower levels than farms C or D.

In our study, the dominant bacterial genera in raw goat milk were *Staphylococcus*, *Escherichia*-*Shigella*, and *Pseudomonas*, with occasional detection of *Lactococcus*, *Corynebacterium*, and *Yersiniaceae*. Previous studies of goat milk microbiota have similarly identified *Staphylococcus* and *Escherichia* as prevalent taxa, largely reflecting contamination from teat skin, fecal matter, or the farm environment [41,42]. By contrast, *Pseudomonas* has been more consistently highlighted as a major psychrotrophic genus in bovine raw milk [43]. These observations suggest that goat and cow milk share certain core taxa, but the relative contribution of spoilage-associated bacteria differs, potentially due to host physiology, teat canal morphology, and farm management practices [44]. Seasonal and lactation-related shifts in milk microbiota have also been documented, which may explain fluctuations in the abundance of dominant taxa [9,45,46]. Notably, some genera reported as dominant in other goat milk studies, such as *Enterococcus*, *Lactococcus*, and *Clostridium* [42,47], were only sporadically detected in our samples. Such discrepancies likely reflect differences in geographic origin, feeding systems, or methodological approaches, and highlight the diversity of goat milk microbiota across production contexts.

Regarding specific microbial genera, *Pseudomonas* species are well known for causing spoilage due to their proteolytic and lipolytic enzyme production and ability to grow at refrigeration temperatures. While *Pseudomonas* is consistently highlighted as dominant in bovine raw milk, in our goat milk samples it was not always abundant; rather, its prevalence appeared strongly influenced by cooling efficiency. Farm A had the lowest relative abundance of *Pseudomonas*, likely due to rapid cooling (to 4 °C in <2 h with an ice bath), whereas the other farms required 2–4 h using standard refrigeration. This pattern supports previous findings that inadequate cooling favors *Pseudomonas* proliferation and accelerates spoilage [48]. Thus, while *Pseudomonas* may not dominate goat milk under all conditions, it can become problematic when cooling is delayed. *Escherichia*-*Shigella* was also frequently detected, consistent with its status as a common genus in goat milk. *Escherichia*-*Shigella* multiplication is often linked to poor hygiene and the presence of feces or organic matter [49], and in our study its relative abundance varied substantially depending on hygiene conditions. In July, farm A had the highest counts, while in August farm B had the lowest. These differences may be explained by parlor design: farm A’s parlor was only partially enclosed, allowing environmental contamination, whereas farm B’s parlor was fully enclosed. This highlights that although *Escherichia-Shigella* is often present, its levels are strongly modulated by hygiene and facility characteristics [50].

*Staphylococcus aureus* was detected in higher relative abundance at farm A compared to farms B and D. This genus is widely recognized as one of the dominant taxa in goat milk [51], but our results indicate that specific farms may show particularly elevated levels. Such variation could be related to handler-associated contamination or subclinical mastitis, though our data do not allow firm conclusions [49,52]. Farm B also showed a relatively higher abundance of *Lactococcus* in August. While this genus was only sporadically detected overall, its increased abundance in Farm B at a specific time point suggests a farm- or feed-related effect. Although generally considered beneficial, *Lactococcus* can also drive rapid acidification and cause undesirable flavors [53]. The elevated levels observed here may reflect feed composition or lactose availability, as SCC values were not consistent with mastitis. Similarly, *Serratia* species were more abundant at farm B in July, though without accompanying udder health issues. *Listeria* species were detected at farm A in July, underscoring the potential role of environmental sources such as soil or manure.

Finally, our regression analysis indicated that hygiene practices significantly shaped the abundance of certain genera. The absence of glove use and hand sanitation was associated with increased levels of *Escherichia*-*Shigella*, *Kocuria*, *Enterococcus*, and *Corynebacterium*, all of which are typically considered fecal- or skin-associated bacteria. Similar associations between poor hygiene and elevated contamination have been reported in dairy cow studies [54,55]. In contrast, several groups, including the genus *Pseudomonas* and the family Yersiniaceae, showed little or no association with hygiene variables, suggesting that they may persist in the farm environment regardless of routine sanitation and are more strongly influenced by factors such as equipment condition or water quality.

This study has several limitations that should be acknowledged. First, the number of farms included was small (*n* = 4) and the sampling period was restricted to July and August. As a result, the findings may not be generalizable across seasons, and further studies covering more diverse farms and longer timeframes are required to validate these observations. Second, the visual assessment of hygiene practices was conducted by a single trained researcher. Although a structured checklist and coding system were applied to minimize subjectivity, the potential for observer bias cannot be excluded. Incorporating multiple independent assessors or inter-rater validation in future studies would strengthen the reliability of such qualitative evaluations. Finally, the week-to-week variability observed in several parameters suggests that additional longitudinal sampling across multiple lactation stages and herd health conditions would be valuable for capturing the full spectrum of variability in raw goat milk microbiota and quality. Beyond these limitations, it is also important to consider how these findings can be implemented across broader contexts. While the present results highlight the role of hygiene and equipment cleaning in shaping milk microbiota, differences in management systems, herd size, and regional practices may influence the applicability of these recommendations in other production settings. Future research should therefore not only validate the findings in larger and more diverse cohorts but also assess strategies for adapting best practices to varying farm infrastructures.

## 5. Conclusions

This study evaluated the milking and cleaning practices of goat dairy farms to understand their impact on the quality and microbiota of raw goat milk. The findings indicate that differences in cleaning and milking procedures both between and within farms affect bacterial counts and the types of microorganisms present in the milk. The results also suggest that practices related to udder and equipment cleaning, as well as the location of the milking parlor relative to the barn, play crucial roles in shaping the milk’s microbiota. Future research should expand to include a larger number of goat dairy farms, investigate the populations of beneficial microorganisms in raw milk, and explore additional milking practices and farm logistics that may influence milk quality. Additionally, testing the implementation of optimized procedures across different farms could provide further insights, such as examining how consistent practices affect milk quality and microbial communities.

## Figures and Tables

**Table 1:** Sampling information for raw milk and environmental sample collection.

Farm	Farm Location	Breed	No. of Milking Does
A	Silverton, OR, USA	American Alpine, La Mancha	6
B	Monroe, OR, USA	Alpine, Oberhasli, Nigerian Dwarf	4
C	Sherwood, OR, USA	American Alpine, La Mancha, Saanen	8
D	Dallas, OR, USA	French and American Alpines	3

**Table 2:** Visual assessment criteria and coding scheme for farm hygiene conditions.

Criterion	Coding Scheme
Barn/parlor layout	0 = milking in barn, 1 = semi-enclosed, 2 = separate enclosed parlor
Barn/parlor cleaning	0 = only occasional sweeping, 1 = regular sweeping, 2 = sweeping and mopping routine
Glove/hand sanitizer use	0 = none, 1 = occasional, 2 = consistent
Udder preparation	0 = minimal (wipes), 1 = moderate (wipes + dip or soap), 2 = intensive (multi-step with agents)
Equipment cleaning	0 = inconsistent/short, 1 = regular but limited, 2 = thorough (hot water or sanitizer with contact)

**Table 3:** Coded results of visual assessment of farm hygiene practices.

Farm	Layout	Barn/Parlor Cleaning	Gloves/Hand Sanitizer	Udder Preparation	Equipment Cleaning
A	1	2	2 (gloves + sanitizer)	2 (iodine/bleach)	2 (bleach + hot wash)
B	2	2	0 (none)	0 (wipes only)	0 (lukewarm, short)
C	2	0	1 (gloves/sanitizer, irregular)	1 (wipes + dip)	2 (dishwasher hot water)
D	0	2	0 (none)	1 (wipes + soap + bleach)	1 (hot water + bleach, short)

**Table 4:** Summary of Kruskal–Wallis and Dunn’s post hoc test results for microbiological and compositional parameters in goat milk across farms.

Parameter	Month	Kruskal–Wallis χ^2^	*p* Value	Significant Pairwise Differences	
				Adjusted *p* (Bonferroni)	Adjusted *p* (FDR: Benjamini–Hochberg)
APC	July	9.36	0.025	A vs. B (0.038), A vs. C (0.028)	A vs. B (0.020), A vs. C (0.028)
APC	August	8.90	0.031	A vs. B (0.010)	A vs. B (0.010)
YM	July	4.11	0.250	None	None
YM	August	7.00	0.072	A vs. B (0.036) *	A vs. B (0.036) *
CC	August	4.11	0.250	None	None
SCC	July	5.15	0.161	None	None
SCC	August	1.97	0.578	None	None
Fat	July	4.33	0.228	None	None
Fat	August	6.28	0.099	None	None
Protein	July	8.08	0.045	A vs. D (0.020)	A vs. D (0.020)
Protein	August	9.46	0.024	A vs. C (0.020)	A vs. C (0.020)
Lactose	July	9.46	0.024	B vs. D (0.020)	B vs. D (0.020)
Lactose	August	8.95	0.030	B vs. D (0.028)	B vs. D (0.028)
Blood NEFA	July	2.59	0.459	None	None
Blood NEFA	August	9.46	0.024	A vs. B (0.007)	A vs. B (0.007)

* Marginal significance in overall test (*p* = 0.072), but pairwise comparison reached significance.

**Table 5:** Associations between hygiene practices and microbial abundance.

Taxa	R^2^	*p* Value	Key Interpretation
*Pseudomonas*	0.305	0.059	Not significant
*Yersiniaceae*	0.307	0.058	Not significant
*Lactococcus*	0.259	0.105	Not significant
*Allorhizobium*	0.298	0.065	Not significant
*Achromobacter*	0.212	0.180	Not significant
*Staphylococcus*	0.330	0.042	Intercept (*p* = 5.59 × 10^−5^)
*Escherichia_Shigella*	0.526	0.002	Intercept (*p* = 9.32 × 10^−7^), Glove use no (*p* = 0.004)
*Deinococcus*	0.428	0.010	Hand sanitation no, Bleach chlorhexidine sanitizer (*p* = 0.005)
*Ochrobactrum*	0.308	0.057	Not significant
*Corynebacterium*	0.351	0.032	Glove use no (*p* = 0.026), Hand sanitation no (*p* = 0.010)
*Acinetobacter*	0.130	0.415	Not significant
*Kocuria*	0.435	0.009	Glove use no, Hand sanitation no (*p* = 0.004)
*Enterococcus*	0.365	0.026	Glove use no (*p* = 0.010), Hand sanitation no (*p* = 0.012)

The intercept represents the condition where glove use = yes, hand sanitation = yes, and sanitizer type = bleach iodine.

## Data Availability

The sequencing data have been deposited in the NCBI Sequence Read Archive (SRA) under accession number SRP584650.
